# Shoplifting as disinhibition in individuals with a borderline pattern

**DOI:** 10.1192/j.eurpsy.2025.1908

**Published:** 2025-08-26

**Authors:** A. Bogdanovska Toskic, S. Arsova Hadzi-Angjelkovska

**Affiliations:** 1 Psychiatry, General Hospital, Kumanovo; 2 University Clinic of Psychiatry, Skopje, North Macedonia

## Abstract

**Introduction:**

Pathological traits according to the dimensional diagnostic system for personality disorders include a certain set of emotional, cognitive and behavioral features. In different people, a certain trait manifests itself in a different behavioral form, while the same trait can manifest itself in several people similarly.

**Objectives:**

To analyze shoplifting in individuals with borderline personality disorder through pathological traits.

**Methods:**

A non-systematized literature review was performed, with keywords: borderline personality disorder, shoplifting, and a case series was presented.

**Results:**

This is a series of three cases of people with borderline personality disorder, who were also assessed according to ICD 11 criteria with LPFS-BF 2.0, PSQ-11 and BPS. The first person is a female, 20 years old, a student, LPFS-BF 2.0 - 34, on PSQ-11 elevated values for all pathological traits, except for anankastia and BPS – 47. She is occasionally depressed and continuously abuses marijuana for a long period of time. The second person is the also female, age 21, student, LPFS-BF 2.0 - 31, on PSQ-11 elevated values for negative affectivity and disinhibition and BPS – 46. Occasionally has obsessive controlling and criticizing thoughts and several episodes of self-harm, incidental substance abuse. The third person is a 20-year-old male, unemployed, LPFS-BF 2.0 - 32, PSQ-11 elevated values for negative affectivity and disinhibition and BPS – 41. He is occasionally paranoid, has a history of self-harm and occasional excessive use of benzodiazepines. All three give information that they occasionally steal from markets and large stores, but they do not steal from people. As a reason for stealing, they cite a lack of funds at a given moment for a particular item they wanted, but also the need to steal, the inability to refrain and the excitement they feel at that moment.

**Conclusions:**

Negative affectivity and disinhibition appear as common pathological traits in all three people. Disinhibition, characterized by impulsivity due to a rush of immediate emotions and thoughts, without thinking about the consequences and the need for immediate gratification, manifests itself in these individuals through shoplifting. Occasional shoplifting, without practice of other forms of stealing, is a disinhibitory phenomenon.

**Disclosure of Interest:**

None Declared

